# Occult breast carcinoma presenting with axillary lymphadenopathy

**DOI:** 10.1186/bcr2992

**Published:** 2011-11-04

**Authors:** MA Crotch-Harvey

**Affiliations:** 1 Macclesfield District General Hospital, Macclesfield, UK

## Introduction

Occult breast carcinoma presenting with axillary lymphadenopathy is an uncommon but difficult clinical problem. The most appropriate diagnostic pathway, the prognosis and the best form of treatment may be uncertain. To answer these questions, we have examined the outcomes of women presenting in this way over a number of years.

## Methods

Thirteen women were identified prospectively over a 12-year period, presenting with suspicious lymphadenopathy but no identifiable breast tumour on initial mammography or ultrasound. Biopsy of the abnormal nodes was consistent with a breast primary in all cases. All women had further imaging with breast MRI (11 cases), breast scintigraphy (one case) and CT scanning of the chest and abdomen. Second-look ultrasound was targeted to suspicious areas identified on second-line imaging. The type of treatment, presence of distant metastases and survival were recorded.

## Results

Further imaging revealed a primary breast lesion in seven cases, six remained truly occult. Follow-up ranged from 3 to 144 months (mean 38 months). Three patients died, one is alive with distant metastases and nine remain disease free. Those with no identifiable primary were treated with chemotherapy usually in combination with radiotherapy.

## Conclusion

The use of MRI and targeted ultrasound-guided biopsy revealed primary tumours in approximately half our cases presenting with lymphadenopathy and negative conventional imaging. Chemotherapy with radiotherapy appears to be an effective treatment for occult breast cancer. The initial staging tests are crucial and if clear the prognosis appears similar to patients with breast cancer and positive axillary nodes.

